# Self-Assembly and Crystal Structure of Boc-Protected Dipeptides Containing L-Phenylalanine and L-Tyrosine

**DOI:** 10.3390/ma19071319

**Published:** 2026-03-26

**Authors:** Rosa M. F. Baptista, Alejandro P. Ayala, Clara S. B. Gomes, Daniela Santos, Michael S. Belsley, Etelvina de Matos Gomes

**Affiliations:** 1 Centre of Physics of Minho and Porto Universities (CF-UM-UP), Laboratory of Physics for Materials and Emerging Technology (LaPMET), University of Minho, Campus de Gualtar, 4710-057 Braga, Portugalbelsley@fisica.uminho.pt (M.S.B.); 2 Departamento de Física, Universidade Federal do Ceará, Fortaleza 65455-900, CE, Brazil; ayala@fisica.ufc.br; 3 i4HB, NOVA School of Science and Technology, NOVA University Lisbon, 2829-516 Caparica, Portugal; clara.gomes@fct.unl.pt

**Keywords:** dipeptides, self-assembly, supramolecular structures, X-ray single crystal structures, optical properties

## Abstract

The self-assembly of a novel synthesized chiral dipeptide, Boc-p-nitro-L-phenylalanyl-tyrosine, into supramolecular structures is investigated by optical absorption and photoluminescence spectroscopy as well as single crystal X-ray diffraction. The compound is a diphenylalanine derivative belonging to a family of aromatic dipeptides that spontaneously self-organize into nanostructures through molecular recognition. The dipeptide exhibits several step-like peaks in its absorption band, indicative of self-assembly into quantum-confined nanostructures. In contrast, the parent Boc-p-nitro-L-phenylalanine amino acid lacks these features, indicating that the tyrosine residue favors quantum-confined self-assembly. Crystal structure determination reveals distinct packing styles: Boc-p-nitro-L-phenylalanine forms two-dimensional hydrogen-bonded layers, while the related p-nitro-free Boc-L-phenylalanyl-tyrosine dipeptide organizes into a 3D helical columnar architecture, driven by the additional hydrogen-bonding capacity of the peptide bond and tyrosine hydroxyl group, which favors the formation of a channel-type tetragonal architecture network over the planar sheets of the monomer. Furthermore, the introduction of a tyrosine residue into the Boc-p-nitro-L-phenylalanine molecule alters its supramolecular assembly, as the dipeptide Boc-p-nitro-L-phenylalanyl-tyrosine crystallizes as a monohydrate. The water molecule present in the structure acts as a bridge, participating in a hydrogen-bonding network between the tyrosine hydroxyl groups of neighboring columns through intermolecular interactions.

## 1. Introduction

The self-assembly of dipeptides has attracted significant attention due to their capacity to form well-defined nanostructures, which hold promising applications in biomaterials, nanotechnology, and energy harvesting. In recent work, there has been an increasing focus on the rational design of these structures to enhance their properties and functionality [1,2,3,4,5,6]. Among these, chiral diphenylalanine-based (Phe-Phe) peptides are of particular interest due to their remarkable ability to self-assemble into a variety of nanostructures, including nanotubes (NT) [7,8,9], nanoribbons [10] and nanospheres (NS) [7,11]. This hierarchical organization is essentially determined by non-covalent interactions—π-π stacking, hydrogen bonding, and van der Waals forces—further influenced by experimental conditions such as pH, temperature, and solvent polarity [12,13]. These nanostructures exhibit exceptional physical properties, particularly in terms of mechanical strength. Phe-Phe nanotubes (NT) are remarkably stiff, with an average point stiffness of 160 N/m and Young’s modulus ranging from 19 to 27 GPa [14]. They are among the stiffest known biological materials. This outstanding rigidity results from “zipper-like” aromatic interlocks that tightly interpenetrate the NT backbones [15]. While Phe-Phe crystallizes in a hexagonal lattice, space group P6_1_ or its enantiomorph P6_5_, N-capped dipeptide, *N*-(*t*-butoxycarbonyl)-L-Phe-L-Phe-COOH (Boc-FF or Boc-Phe-Phe, Boc = *N*-(*t*-butoxycarbonyl)) crystallizes in a monoclinic lattice, space group C2. This symmetry lowering due to the molecular addition of the Boc group results in a Young’s modulus that is higher than that of Phe-Phe and is accompanied by higher mechanical anisotropy and flexibility [16].

In this context, we have previously investigated the self-assembly properties of several Boc-protected chiral dipeptides: Boc-L-phenylalanyl-L-phenylalanine (Boc-Phe-Phe) [17], Boc-L-phenylalanyl-L-tyrosine (Boc-Phe-Tyr) [18], Boc-L-phenylalanyl-L-phenylalanine-benzothiazole (Boc-Phe-Phe-Bz) [19] and Boc-*p*-nitro-L-phenylalanyl-*p*-nitro-L-phenylalanine (Boc-pNPhe-pNPhe) [18,20,21]. These derivatives of diphenylalanine have also demonstrated the capacity to form self-assembled nanostructures with quantum confinement. Optical absorption measurements have revealed the presence of bands with subpeaks characteristic of quantum-confined structures for all dipeptides. The Boc-pNPhe-pNPhe crystal structure was recently determined and assigned to a monoclinic lattice, space group P2, and its optical second harmonic generation response was studied [20].

Phenylalanine (Phe) and tyrosine (Tyr) are structurally similar amino acids, differing by the presence in Tyr of a phenolic hydroxyl (-OH) group at the *para* position of the benzene ring, which plays a crucial role in intermolecular π-π stacking, and, along with hydrogen bonding, facilitates gel formation. Perween et al. have shown that aromatic amino acids, such as Phe and Tyr, do in fact form distinct fibrillar structures under neutral, aqueous conditions [22]. The additional hydroxyl group in Tyr preferentially engages in intermolecular hydrogen bonding rather than π-π stacking. Singh et al. report self-assembly of the aromatic amino acid Tyr in dimethylsulfoxide (DMSO), forming a supramolecular gel characterized by nanofibrils, driven by noncovalent interactions such as hydrogen bonding and π-π stacking among Tyr molecules [23]. Nanotubes were also synthesized by Babar and Sakar using a simple building block, the Tyr amino acid, through self-assembly, employing ethanol as a solvent [24].

The Tyr functional group significantly influences the self-assembly and properties of peptide-based systems [25]. Beyond its role in molecular conformation, Tyr also enhances charge transport through proton-coupled electron transfer reactions, contributing to the stability and functionality of supramolecular nanostructures [26]. A study conducted by Bera et al. investigates how tyrosine-modified diphenylalanine analogs self-organize differently: the peptide Boc-Phe-Phe-OMe forms twisted fibrils while the tyrosine-modified analogues (Boc-Phe-Tyr-OMe, Boc-Tyr-Phe-OMe, and Boc-Tyr-Tyr-OMe) form microspheres [27].

In this work, we report the synthesis and investigate the self-assembly of the new chiral dipeptide Boc-*p*-nitro-L-phenylalanyl-tyrosine (Boc-pNPhe-Tyr) into supramolecular structures, highlighting the impact of functional groups on their chemical and structural properties. The optical properties of the Boc-pNPhe-Tyr dipeptide and its parent amino acid Boc-*p*-nitro-L-phenylalanine (Boc-pNPhe) are compared with those of the Boc-Phe-Tyr dipeptide, addressing the contribution of the Tyr residue on their molecular organization and self-assembly behavior at the nanoscale.

The introduction of the electron-withdrawing nitro group (-NO_2_) makes phenylalanine electron-deficient, enhancing its ability to form stable self-assembled structures, such as fine fibrils with high aspect ratios [28,29], and microtapes composed of self-assembled nanotubes [18,20]. This modification promotes greater rigidity in supramolecular structures due to a combination of π-π, dipole–dipole, and van der Waals interactions, which contribute to the stabilization of ordered nanostructures, improving their optical properties. Also, the Boc protection group plays a role in modulating solubility and steric hindrance, further influencing the self-assembly process [30].

In addition, single-crystal X-ray diffraction is used to determine the crystal structures of the amino acid Boc-pNPhe and the two dipeptides Boc-Phe-Tyr and Boc-pNPhe-Tyr, which were not previously known. The combination of structural and spectroscopic analysis contributes to an understanding of the depth of self-assembly and potential applications in material science areas such as energy harvesting or biotechnology, as have been reported for related dipeptides [2,5,10,13,16,17,19]. The new dipeptide Boc-p-nitro-L-phenylalanyl-tyrosine is chiral, lacking a center of symmetry, which allows for the existence of piezoelectricity and optical second harmonic generation properties, as have previously been reported for diphenylalanine and other family derivative dipeptides.

## 2. Materials and Methods

### 2.1. Materials

*p*-Nitro-L-phenylalanine (pNPhe), L-tyrosine (Tyr), 1-hydroxybenzotriazole (HOBt), *N*,*N*-dicyclohexylcarbodiimide (DCC), thionyl chloride, and di-*tert*-butylpyrocarbonate (Boc_2_O) were purchased from Merck/Sigma-Aldrich (Darmstadt, Germany), Alfa Aesar, and TCI (all purchased from Cymit Química, Barcelona, Spain, the distributor for both brands) and used as received. The solvents employed in this study, including dichloromethane (DCM), 1,1,1,3,3,3-hexafluoro-2-propanol (HFP), ethanol, methanol, ethyl acetate and *N*,*N*-dimethylformamide (DMF), were purchased from Merck/Sigma-Aldrich. 1,4-dioxane was purchased from Fisher Chemicals (Zurich, Switzerland). All solvents were used as received, without further purification.

### 2.2. Synthesis of Boc-p-nitro-L-phenylalanyl-L-tyrosine (Boc-pNPhe-Tyr)

**Figure 1:**
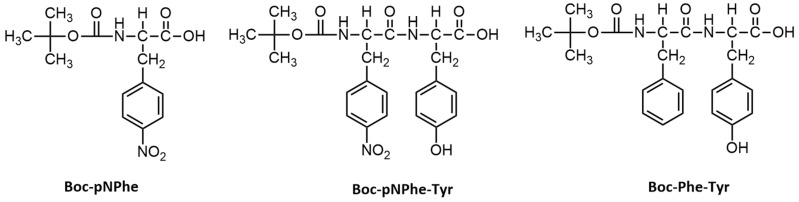
Molecular structures of the studied amino acid and dipeptides.

A reaction of the commercially available L-amino acid Tyr with thionyl chloride in methanol was performed to give the methyl ester of the corresponding amino acid (Tyr-OMe). Simultaneously, p-nitro-L-phenylalanine (pNPhe) and L-tyrosine (Tyr) were reacted with di-*tert*-butylpyrocarbonate (Boc_2_O) in 1,4-dioxane at 0 °C, affording the N-Boc-protected amino acids Boc-p-nitro-L-phenylalanine (Boc-pNPhe), obtained as a white solid, and Boc-L-tyrosine (Boc-Tyr), obtained as an oil. The mixture of amino acids in dioxane (40 mL), water (20 mL), and NaOH 1 M (20 mL) was stirred and cooled in an ice bath. Di-*tert*-butylpyrocarbonate (4.8 g) was added, and stirring continued for 6 h. The solution was concentrated under vacuum, cooled, covered with ethyl acetate (50 mL), and acidified with KHSO_4_ to pH 2–3. The aqueous phase was extracted with ethyl acetate, and the extracts were pooled, washed with water, dried over anhydrous Na_2_SO_4_, and evaporated under vacuum. The dipeptide derivative Boc-pNPhe-Tyr was synthesized by solution phase synthesis according to a previously described procedure [17,31], mediated by DCC/HOBt. Briefly, 1.0 equiv. of Boc-pNPhe was dissolved in anhydrous dichloromethane (DCM), and the solution was cooled to 0 °C using an ice bath. Tyr-OMe (1.0 equiv.) was obtained by neutralizing the methyl ester hydrochloride, extracting with ethyl acetate, and concentrating. The solution was added to the reaction, followed by DCC (1.2 equiv.) and HOBt (1.2 equiv.). The mixture reacted for 48 h at room temperature. After DCM evaporation, the residue was dissolved in ethyl acetate, filtered to remove DCU (dicyclohexylurea, a common byproduct in peptide coupling reactions that use DCC as an activation reagent), and washed sequentially with 2 M HCl, brine, 1 M Na_2_CO_3_, and brine. After drying and evaporation, Boc-pNPhe-Tyr-OMe was obtained as a white solid. The methyl ester group was then deprotected with NaOH (2M), and the progress of saponification was monitored by thin-layer chromatography (TLC). After 10 h, methanol was removed under vacuum, and the residue was dissolved in water, washed with DCM, and extracted with ethyl acetate after pH adjustment. The extracts were dried and evaporated under vacuum, yielding Boc-pNPhe-Tyr as a white solid (67%). Additional experimental details, including the structural characterization by NMR spectroscopy, are provided in the Supplementary Information (Section S1.1). Figure 1 shows the molecular structures of the studied dipeptides and amino acids. The synthesis of Boc-Phe-Tyr was reported before in [18].

### 2.3. Self-Assembly of Dipeptide Nanostructures in HFP/Water and Ethanol/Water Solutions

Solutions of Boc-pNPhe and Boc-pNPhe-Tyr were prepared by dissolving the amino acid and the dipeptide in a 2 mg/mL concentration in HFP/water (0.2/9.8 *v*/*v*). The solutions were left at room temperature for 24 h to allow self-assembly to occur. After that, a few drops of both solutions were placed on silica slides, and the solvent was removed by slow evaporation at room temperature before the samples were subjected to SEM analysis.

### 2.4. Optical Absorption and Photoluminescence

Optical absorption (OA) measurements of Boc-pNPhe (0.06–0.3 mM) and Boc-pNPhe-Tyr (0.001–0.06 mM) solutions in ethanol were carried out using a Shimadzu UV-3101PC spectrophotometer (Shimadzu, Kyoto, Japan) operating across the UV–vis–NIR range. Photoluminescence (PL) measurements were carried out using a Fluorolog 3 spectrofluorimeter (HORIBA Jobin Yvon IBH Ltd., Glasgow, UK). Samples were placed in a quartz cuvette with a 1 cm optical path length. Normalized PL spectra of Boc-pNPhe (0.1 mM in ethanol) were recorded using excitation wavelengths ranging from 279 to 360 nm, with both excitation and emission slits fixed to ensure a spectral resolution of 4 nm. For Boc-pNPhe-Tyr (0.06 mM in ethanol), excitation wavelengths from 275 to 360 nm were employed.

### 2.5. Scanning Electronic Microscopy (SEM)

The morphology, diameter distribution, and thickness of the dipeptide nanostructures were quantified using a Nova NanoSEM scanning electron microscope (Thermo Fisher Scientific Inc., Waltham, MA, USA), at an accelerating voltage of 10 kV. Dipeptide single crystals were deposited on a silica surface that had previously been covered with a thin film (10 nm thickness) of Au-Pd (80–20 weight %) using a high-resolution sputter cover, 208HR (Cressington Company, Watford, UK), coupled with an MTM-20 Cressington high-resolution thickness controller. The diameter range of the produced microstructures was measured from SEM images using the ImageJ 1.51n image processing software (NIH, https://imagej.net/ij/, accessed on 10 March 2025). The average diameter and diameter distribution were determined by measuring a number of random nanofibers from the SEM images. Statistical analysis was performed using the OriginPro 2017 SR2 software (OriginLab Corporation, USA), and fiber diameter distributions were fit to a log-normal function.

### 2.6. Single Crystal X-Ray Diffraction (SCXRD)

Single crystals of Boc-pNPhe, Boc-Phe-Tyr and Boc-pNPhe-Tyr that were suitable for X-ray diffraction were mounted on MiTeGen micromounts with immersion oil. Diffraction data were collected on a Bruker D8 VENTURE dual wavelength Mo/Cu CPAD κ-geometry diffractometer equipped with a PHOTON II CPAD area detector. The instrument utilizes microfocus sealed X-ray tubes and multilayer mirrors to ensure monochromaticity.

Data collection and an initial cell determination were performed using the APEX5 software suite [32]. Data reduction and integration were carried out with SAINT [33], and a multi-scan absorption correction was applied using SADABS [34].

The structures were solved by intrinsic phasing using SHELXT [35] and refined by full-matrix least-squares on F^2^ using SHELXL [36], integrated within the Olex2 interface [37] or in WinGX-Version 2021.3 [38] (for Boc-pNPhe-Tyr). All non-hydrogen atoms were refined with anisotropic displacement parameters. Hydrogen atoms were placed in idealized positions and refined using a riding model. For Boc-Phe-Tyr, the SQUEEZE routine within PLATON [39] was used to treat a disordered solvent that could not be modeled with discrete atoms. The sample of Boc-pNPhe-Tyr was of poor quality, showing long needle-shaped crystals with low diffracting power, which led to a high Rint value. Nevertheless, the structure refined to convergence and it was possible to establish its molecular structure, which agrees with that obtained through other analytical techniques. Crystallographic data have been deposited with the Cambridge Crystallographic Data Centre (CCDC) under deposition numbers 529055 (Boc-pNPhe), 529054 (Boc-Phe-Tyr), and 2530636 (Boc-pNPhe-Tyr), and are available via www.ccdc.cam.ac.uk/structures (accessed on 10 March 2025).

## 3. Results and Discussion

### 3.1. Self-Assembly of Boc-Protected Amino Acid and Dipeptide

**Figure 2:**
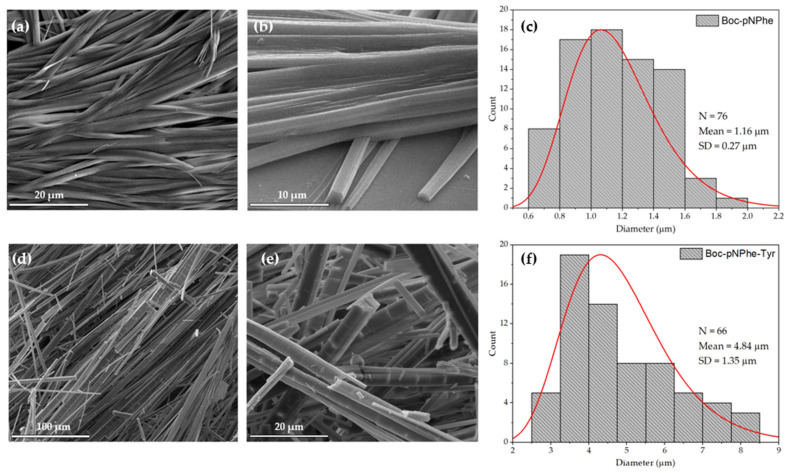
SEM micrographs with distribution diameter of self-assembled Boc-pNPhe (**a**–**c**) and Boc-pNPhe-Tyr (**d**–**f**) structures at 2 mg/mL concentration in HFP/water (0.2/9.8 *v*/*v*), at magnification levels 1000×, 5000× and 10,000×, and their thickness distribution. The red line shows a fit Log-Normal distribution.

Figure 2 demonstrates the self-assembly of Boc-pNPhe amino acid and Boc-pNPhe-Tyr dipeptide at a concentration of 2 mg/mL in HFP/water (0.2/9.8 *v*/*v*). As illustrated in Figure 2a,b, Boc-pNPhe generates ribon-like structures, with a paralelipidec morphology (some tending to be more cylindrical), with an average thickness of 1.16 µm and lengths of tens of microns. Boc-pNPhe-Tyr forms structures with similar morphology but a higher average thickness of 4.84 µm, related to the formation of a bigger molecule crystallizing in a larger unit cell. This is shown in Figure 2d,e, consistent with the formation of ordered self-assembled structures, as previously reported for other family members [18,19,40,41].

Self-assembly studies reported on Tyr water solutions showed that these amino acid aggregates form flat ribbon-like structures with a textural morphology and dimensions of up to 2 μm in width and several micrometers in length [22]. Furthermore, when deposited onto different surfaces, Tyr can self-assemble into nanoribbons, branched structures and fern-like supramolecular architectures. It was reported that for several peptide sequences, the presence of a Tyr residue will drive the self-assembly through supramolecular recognition, controlling the structure formation process [42]. However, our results suggest that Tyr residue does not significantly alter the self-assembly morphology of Boc-pNPhe when Boc-pNPhe-Tyr is formed, while the presence of *p*-nitro drives its morphology. From crystallography, it is known that unit cell crystal symmetry determines the crystal morphology, which explains why, in this case, the morphologies are similar, as both compounds crystallize in point group 2, chiral space group P2_1_ (Section S1.2).

Furthermore, it is interesting to mention that Boc-p-nitro-L-phenylalanyl-p-nitro-L-phenylalanine dipeptide, where Tyr residue has been substituted by a nitro-phenylalanine residue, undergoes a dual self-assembling process from HFP/water solutions, where the self-assembly of micro tapes results from the self-assembly of nanotubes [18], demonstrating the strong influence of the *p*-nitro group.

### 3.2. Optical Absorption and Photoluminescence

The photophysical properties of self-assembled Boc-pNPhe amino acid and Boc-pNPhe-Tyr dipeptide dissolved in ethanol were evaluated by measuring, at 298 K, their optical absorption (OA) and photoluminescence (PL) spectra. Quantum confinement (QC) manifests itself in dipeptides through the presence of either spike-like or step-like peaks over the OA spectra. They are determined by the formation of electron–hole pairs with strong Coulomb binding energy, indicating the formation of quantum dots (QD) or two-dimensional quantum wells (2D QW) inside the self-assembled structures, as proposed before for diphenylalanine and its family of related dipeptides [9,17,43,44,45,46].

**Figure 3:**
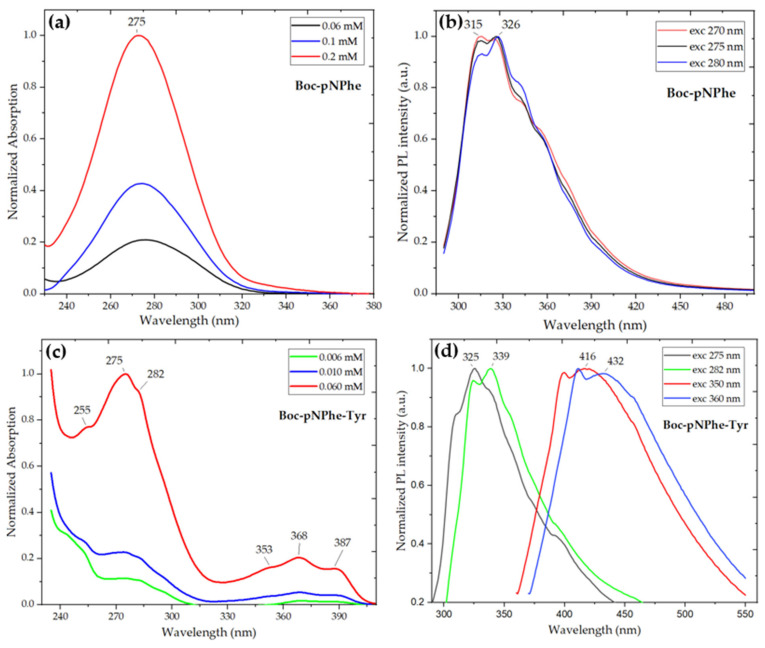
Optical absorption (OA) and photoluminescence (PL) spectra of Boc-pNPhe amino acid and Boc-pNPhe-Tyr dipeptide, dissolved in ethanol: (**a**,**c**) Normalized OA spectra of the amino acid and dipeptide at different concentrations, with the last exhibiting three step-like peaks over each of the absorption bands. (**b**) Normalized PL spectra of Boc-pNPhe, with a concentration of 0.1 mM, at different excitations ranging between 270 and 280 nm. (**d**) Normalized PL spectra of Boc-pNPhe-Tyr, with a concentration of 0.06 mM, at different excitations ranging between 275 and 360 nm.

Figure 3 shows the measured OA and PL spectra of the self-assembled nanostructures formed. In Boc-pNPhe Figure 3a, the three OA spectra, corresponding to increasing concentrations from 0.06 mM to 0.2 mM, show a single broad absorption band between 240 and 320 nm, with a maximum absorption peak centered at 275 nm. Quite differently, Figure 3c shows that for Boc-pNPhe-Tyr, the OA spectra exhibit two distinct bands: one more intense band centered between 240 and 320 nm, and a less intense band centered between 330 and 395 nm. Furthermore, the OA spectrum displayed by Boc-pNPhe-Tyr shows three step-like peaks located at 255 nm (4.82 eV), 275 nm (4.51 eV), and 282 nm (4.40 eV) for the first OA band, with wavelength differences in the range of 18–7 nm (energy difference of 0.31–0.11 eV) between adjacent peaks. For the second OA band, the step-like peaks occur at 353 nm (3.51 eV), 368 nm (3.37 eV), and 387 nm (3.2 eV) with wavelength differences of 15–11 nm (energy difference of 0.14–0.17 eV), the intensity of which increases with the dipeptide concentration, indicative of the formation of a larger number of nanostructures. The observation of this second OA band invites a future detailed investigation into the optical properties and self-assembly of this dipeptide. Importantly, those step-like features over the OA band are consistent with the existence of QC, which has been reported before for Phe-Phe nanotubes and several derivative dipeptides [7,17,18,19,21,43,47,48,49,50].

The OA spectrum of Boc-pNPhe amino acid does not show those step-like peaks, indicating that the introduction of the tyrosine residue in the dipeptide promotes significant changes in supramolecular organization, favoring the formation of ordered domains with discrete electronic features typical of quantum-confined self-assembled nanostructures. These results are in agreement with previous studies demonstrating the crucial role of tyrosine in guiding and stabilizing peptide self-assembly due to its ability to establish intermolecular hydrogen bonds, as well as additional π–π interactions provided by its phenolic ring [51,52]. To gain a better insight into the importance and influence of the Tyr residue on QC, we should recall that the OA spectra of Boc-Phe-Tyr also showed six step-likes peaks, but in that case these were located at 252 nm (4.92 eV), 258 nm (4.81 eV), 265 nm (4.68 eV), 269 nm (4.61 eV), 278 nm (4.46 eV) and 285 nm (4.35 eV) over a band centered between 250 and 320 nm (for this dipeptide there was no second OA band at longer wavelengths) [18]. However, Boc-Phe-Phe instead showed four characteristic spike-like peaks, located at 248 nm, 253 nm, 259 nm and 265 nm, which were already observed in the OA of the Phe-Phe dipeptide and are indicative of the formation of QD in self-assembled dipeptide structures [17]. Furthermore, when the Tyr in Boc-pNPhe-Tyr is substituted by pNPhe residue, forming a Boc-pNPhe-pNPhe dipeptide, those step-like peaks are present at 268, 272, 275, 279 nm, but are much less pronounced [18]. We conclude that while for Boc-Phe-Phe and Phe-Phe, four spike-like peaks exist over the OA band, substitution of Phe by Tyr residue provokes an increase in the number of subpeaks, which now take a step-like form, consistent with the formation of QW structures [43,45]. This feature has previously been reported for a Phe-Tyr dipeptide [9]. Table 1 summarizes these results.

The PL spectra for Boc-pNPhe in Figure 3b, obtained with excitations at three different wavelengths, 270 nm, 275 nm and 280 nm, lie in the range of 280 nm to 480 nm with a maximum shifting from 315 nm to 326 nm within the UVB region of the electromagnetic spectrum. However, the presence of tyrosine in the Boc-pNPhe-Tyr dipeptide system originates a different PL spectrum, formed by two pairs of close emissions, as shown in Figure 3d: the first pair is in the range of 300–450 nm with maxima at 325 nm and 339 nm; the second pair of emissions is in the range of 360–550 nm with maxima at 416 nm and 432 nm, within the blue (visible) spectral range of the electromagnetic spectrum. This means that by tuning the excitation wavelength from 275 nm to 360 nm, one can obtain a broad PL response ranging from 300 nm to 550 nm, which may be useful in energy transfer radiation processes where a large spectral overlap area is a key feature of great importance [53,54].

The photoluminescence (PL) emission of the Boc-Phe-Tyr dipeptide under excitation at 278 nm was previously analyzed in HPF/ethanol and HPF/water solutions [18]. It was observed that, although the emission maximum remains at the same wavelength, its intensity decreases with increasing concentration. This behavior is associated with the narrowing of the exciton peak. In more dilute solutions, where ordered structures are not formed, the PL intensity is higher, whereas in more concentrated solutions, the formation of ordered nanostructures leads to reduced PL emission [50].

Further studies on dynamic light scattering under different experimental conditions, such as temperature, solvents and pH, need to be addressed to deepen the understanding of the self-assembly process. Growing suitable crystals under those conditions will establish a fuller comprehension of the subject.

## 4. X-Ray Crystal Structures of the Amino Acid and Dipeptides

### 4.1. Crystal Structure of Chiral Boc-pNPhe Amino Acid

**Figure 4:**
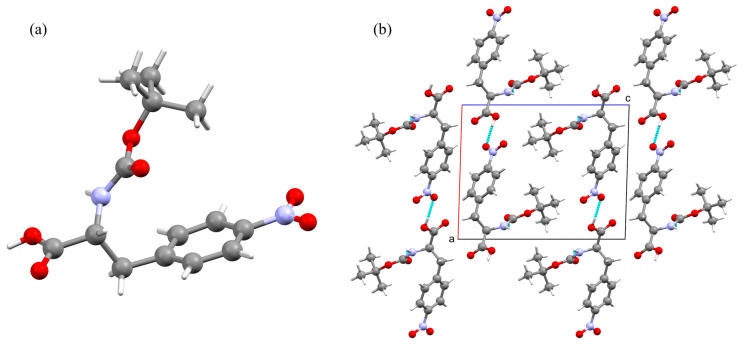
(**a**) View of the asymmetric unit of Boc-pNPhe. (**b**) Projection of the crystal packing onto the crystallographic ac plane.

Boc-pNPhe crystallizes in the non-centrosymmetric monoclinic space group P2_1_ with unit cell parameters a = 10.7575(6) Å, b = 5.1460(3) Å, c = 13.4066(8) Å, and β = 91.739(4)°. Crystallographic data and structure refinement details are summarized in Table S1. XRD-Data: The asymmetric unit contains one molecule with bond lengths and angles that fall within expected ranges (Figure 4). The conformation of the amino acid backbone is defined by the torsion angles (N1–C8–C9–O3) of −155.2(8)° and C11–N1–C8–C7 of −99.7(8)°, while the side chain adopts a gauche+ conformation characterized by an angle (N1–C8–C7–C4) of 52.6(9)°. The tert-butoxycarbonyl (Boc) protecting group maintains a trans geometry across the urethane linkage, evidenced by the torsion angle (C12–O6–C11–N1) of 172.2(6)°. The 4-nitro substituent is nearly coplanar with the phenyl ring, with an O1–N10–C1–C6 torsion angle of −0.5(12)°, indicating effective conjugation between the nitro group and the aromatic system.

This structure can be compared with that of the parent amino acid, L-4-nitrophenylalanine monohydrate [55]. Unlike the Boc-protected derivative, which exists as a neutral carboxylic acid, the free amino acid crystallizes as a zwitterion. This difference in ionization state leads to a distinct supramolecular arrangement: while Boc-pNPhe relies on conventional O-H⋯O and N-H⋯O hydrogen bonds to form 2D layers, the parent amino acid utilizes charge-assisted hydrogen bonds (N-H(+)⋯O(−)) between the ammonium and carboxylate groups to stabilize its lattice. Furthermore, the side-chain conformation differs significantly; Boc-pNPhe adopts a gauche+ conformation, whereas the free amino acid exhibits an anti (trans) conformation, likely driven by the different packing requirements imposed by the zwitterionic network and the absence of the bulky Boc group.

The supramolecular architecture of Boc-pNPhe is organized into a two-dimensional network extending parallel to the ab plane, stabilized by intermolecular hydrogen bonding (Table S2). A strong hydrogen bond connects the carboxylic acid hydroxyl group to a nitro group oxygen atom of a neighboring molecule (O4-H4⋯O1), with a donor–acceptor distance of 2.807(9) Å. This interaction is complemented by a hydrogen bond between the carbamate nitrogen and the carbonyl oxygen of the Boc group in an adjacent molecule (N1-H1⋯O5), exhibiting a distance of 3.063(9) Å.

Despite the presence of phenyl rings, no significant π-π stacking is observed, as the centroid-to-centroid distance between parallel rings is approximately 5.15 Å, with a slippage of 4.22 Å. This distance precludes direct orbital overlap. Instead, the aromatic packing is stabilized by T-shaped C-H⋯pi interactions. The polarized aromatic protons of the p-nitrophenyl ring serve as donors to the electron-rich pi-systems of adjacent molecules, forming a “herringbone-like” aromatic zipper that stabilizes the hydrophobic layers in the ac plane.

### 4.2. Crystal Structure of Chiral Boc-Phe-Tyr Dipeptide

**Figure 5:**
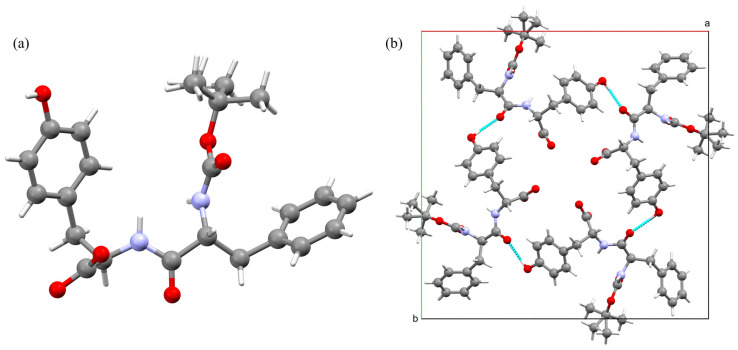
(**a**) View of the asymmetric unit of Boc-Phe-Tyr. (**b**) Projection of the crystal packing onto the crystallographic ab plane.

Boc-Phe-Tyr crystallizes in the tetragonal space group P4_3_, with unit cell parameters a = b= 21.2134(13) Å, c = 5.1784(5) Å, and Z = 4. Crystallographic data and structure refinement details are summarized in Table S1. The structure is characterized by a notably short c-axis of approximately 5.18 Å, which corresponds to the translation distance between stacked dipeptide molecules. This periodicity is a characteristic feature of amyloid-like cross-beta assemblies and related self-assembling peptides, indicating that the molecules stack effectively along the c-axis. The asymmetric unit contains one molecule of Boc-Phe-Tyr (Figure 5). The peptide backbone adopts an extended conformation, facilitating the formation of intermolecular hydrogen bonds perpendicular to the stacking axis. The packing reveals the formation of continuous channels running along the c-axis, a common feature in peptide assemblies crystallizing in chiral tetragonal or hexagonal space groups.

Consistent with the porous nature of the framework, these channels contained disordered solvent molecules, which were treated using the SQUEEZE procedure [39]; specifically, a void volume of 37 Å^3^ was found to contain 15 electrons, corresponding to disordered water molecules.

The supramolecular organization of Boc-Phe-Tyr is governed by a robust network of classical (N-H⋯O, O-H⋯O) and weak (C-H⋯O) hydrogen bonds that operate orthogonally to stabilize the 3D architecture (Table S3). The primary driving force for crystallization is the infinite chain of hydrogen bonds running along the c-axis. The urethane N-H donor forms a strong interaction with the urethane carbonyl oxygen of the adjacent molecule (N2-H2⋯O5). The bond length is 2.950(10) Å. This directional interaction is responsible for the characteristic 5.18 Å translation observed in the unit cell, effectively stacking the dipeptides into continuous “amyloid-like” columns.

While the stacking is driven by the backbone, the 3D lattice is knitted together laterally by the side chains. The tyrosine hydroxyl group acts as a donor to the peptide carbonyl oxygen of a symmetry-related molecule (O1-H1A⋯O4). This short and linear bond (2.729(10) Å) links the helical columns, creating a rigid tetragonal framework. A unique feature of this structure is the cooperative role of weak C-H⋯O interactions. The aromatic C11-H11 donor (from the tyrosine ring) binds to the same peptide carbonyl acceptor (O4) utilized by the strong hydroxyl interaction (C11-H11⋯O4, d = 3.25 Å). This results in a bifurcated motif where the tyrosine side chain is doubly anchored to the peptide backbone of the neighboring assembly. This cooperative locking mechanism likely restricts the rotational freedom of the tyrosine ring and could contribute to a greater mechanical stiffness of the crystal compared to structures relying solely on single-point hydrogen.

The translation of peptide monomers along the 5.18 Å c-axis places the aromatic side chains of phenylalanine and tyrosine at a distance too great for direct π-stacking (>5 Å). Consequently, the stabilization of the aromatic domains is driven by lateral C-H⋯π interactions between the side chains of neighboring columns. These interactions serve as interlocking bridges, interconnecting the tubular assemblies into a cohesive 3D bundle. The supramolecular assembly is further reinforced by weak C-H⋯O hydrogen bonds and C-H⋯π interactions. The C11-H11⋯O4 interaction (d = 3.25 Å) supports the primary helical motif, acting cooperatively with the strong O1-H1A⋯O4 bond to lock the tyrosine side-chain conformation.

It is particularly insightful to compare the dipeptide structure with that of the monomeric precursor, Boc-pNPhe. Despite the chemical modification—substitution of the para-nitro group with a tyrosine residue via a peptide linkage—the N-terminal Boc-Phe- segment retains a geometric configuration remarkably similar to that observed in the Boc-pNPhe monomer. However, the introduction of the tyrosine moiety drastically alters the supramolecular assembly. While the monomeric Boc-pNPhe self-assembles into 2D hydrogen-bonded layers via acid–nitro and amide–boc interactions, the dipeptide Boc-Phe-Tyr organizes into a 3D helical columnar architecture. This structural shift is driven by the additional hydrogen-bonding capacity of the peptide bond and the tyrosine hydroxyl group, which favors the formation of a tetragonal network over the planar sheets of the monomer.

The structure can also be compared with the well-known Phe-Phe dipeptide. While that one forms hexagonal nanotubes or flexible layered crystals, depending on solvent conditions [16], Boc-Phe-Tyr adopts a tetragonal symmetry. Both systems share the propensity to form channel-type architectures with a characteristic ~5.2 Å stacking axis. However, the presence of the phenolic hydroxyl group in the tyrosine residue of Boc-Phe-Tyr likely introduces specific lateral hydrogen bonding interactions that break the hexagonal symmetry typical of the Phe-Phe motif, resulting in the observed four-fold helical arrangement. This suggests that the Phe-to-Tyr substitution preserves the tubular packing tendency but modulates the symmetry and pore characteristics of the resulting material.

### 4.3. Crystal Structure of Chiral Boc-pNPhe-Tyr Dipeptide

**Figure 6:**
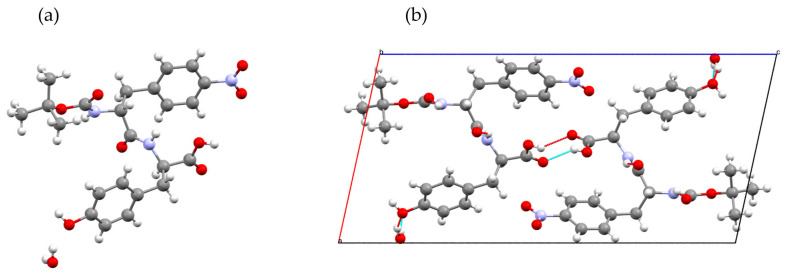
(**a**) View of the asymmetric unit of Boc-pNPhe-Tyr. (**b**) Projection of the crystal packing onto the crystallographic ac plane.

The dipeptide derivative Boc-pNPhe-Tyr crystallizes as a monohydrate in the non-centrosymmetric monoclinic space group *P*2_1_, with unit cell parameters a = 11.050(2) Å, b = 5.0136(9) Å, c = 22.712(4)Å, and Z = 2. Crystallographic data and structure refinement details are summarized in Table S1 (Supporting Information, SI). The asymmetric unit consists of one molecule of Boc-pNPhe-Tyr and one water molecule of crystallization (Figure 6).

Like its non-nitrated analog (Boc-Phe-Tyr), the peptide backbone adopts a notably extended conformation. This “stretched” geometry is essential for the formation of the intermolecular hydrogen-bonding network that defines its supramolecular behavior. The *para*-nitro substitution on the phenylalanine ring adds significant electron-withdrawing character to the *N*-terminal side chain, though it does not fundamentally disrupt the peptide’s ability to adopt the extended β-strand-like state.

This structure exhibits a feature that mirrors the amyloid-like behavior observed in Boc-Phe-Tyr in the short crystallographic b-axis of 5.01 Å, which corresponds to the translation distance between stacked molecules. In this monoclinic lattice, the molecules stack head-to-tail along the b-axis to create continuous columns. This periodicity is a characteristic of cross-beta architectures, in which the peptide backbones are aligned to maximize hydrogen bonding between adjacent units.

The 3D-supramolecular arrangement of Boc-pNPhe-Tyr is controlled by a set of both classical (N-H⋯O and O-H⋯O) and non-classical (C-H⋯O) hydrogen bonds (Table S4, SI). The main driving force for crystallization is the establishment of N-H⋯O intermolecular hydrogen bonds, as the amide N-H groups act as donors to the carbonyl oxygens of the molecule translated by exactly one unit cell. This creates the infinite chain that supports the columnar stacking. Additionally, the hydroxyl group of the carboxylic acid (O6-H6) acts as a strong hydrogen bond donor. Rather than forming a simple dimer, as is common for isolated carboxylic acids, it interacts with the carbonyl oxygen (O7) of a related molecule, playing a dual role in stabilizing the supramolecular architecture. This is primarily achieved by anchoring the columns laterally and establishing a chain-like connection between molecules related by the screw axis symmetry operation: −x + 1, y − 1/2, −z + 1. This ability to cross-link the columns is relevant because it ensures the formation of a rigid, long-range ordered crystal through the specific O-H⋯O interaction with adjacent molecules. Without this interaction, we postulate the dipeptide would form flexible fibers or disordered gels instead of an ordered crystalline structure. Furthermore, the phenolic hydroxyl group in the tyrosine residue serves as a critical lateral anchor. By donating a hydrogen bond to a symmetry-related acceptor in an adjacent column, it reinforces the pillars to form a cohesive 3D crystal.

The Boc-pNPhe-Tyr dipeptide crystallizes as a monohydrate. Unlike the anhydrous or SQUEEZE-treated pores of related structures, the water molecule present in this crystal structure plays a structural role. It acts as a bridge, participating in a hydrogen-bonding network between the tyrosine hydroxyl groups (O8) of neighboring columns through the intermolecular interactions O9-H109⋯O8 (within the asymmetric unit) and O9-H209⋯O8 with symmetry-related molecules (x,y − 1,z).

Introducing the *p*-nitro group onto the phenylalanine ring significantly alters its electronic properties compared to the standard Boc-Phe-Tyr. While the backbone maintains its columnar stacking, the nitro group introduces the potential for new weak interactions. In the *P*2_1_ lattice, the nitro oxygens act as a weak acceptor for C–H⋯O interactions from the neighboring CH_2_ substituent (C18–H18A⋯O4), which provides additional stabilization to the supramolecular structure. Compared to the Boc-Phe-Tyr structure (tetragonal *P*4_3_), the addition of the nitro group and the presence of a stoichiometric water molecule appear to induce lower symmetry in the final crystal structure, changing it from tetragonal to monoclinic. Additionally, Boc-Phe-Tyr forms a four-fold helical arrangement with large solvent channels, whereas a two-fold screw arrangement is observed in Boc-pNPhe-Tyr. Although the Phe-to-pNPhe substitution maintains a tubular, columnar tendency, the nitro substituent and the associated water molecule favor a denser, more compact monoclinic arrangement than the porous tetragonal structure of the unsubstituted dipeptide. This suggests that the nitro group can be used as a symmetry-breaker to transform the structure from a channel-type to a more tightly packed crystalline lattice.

## 5. Conclusions

The optical absorption spectrum (OA) of the novel Boc-*p*-nitro-L-phenylalanyl-tyrosine (Boc-pNPhe-Tyr) dipeptide exhibits two distinct bands, each one displaying several step-like peaks located over them. This feature reveals self-assembly into nanostructures with quantum confinement, a characteristic of the diphenylalanine and derivative dipeptides family. Differently, the OA of the Boc-*p*-nitro-L-phenylalanine (Boc-pNPhe) amino acid does not show step-like peaks, indicating that the introduction of the tyrosine residue favors the formation of quantum-confined self-assembled nanostructures.

The supramolecular architecture of Boc-pNPhe is organized into a two-dimensional network extending parallel to the ab plane, stabilized by intermolecular hydrogen bonding. It crystallizes in the chiral monoclinic space group P2_1_ with no significant π-π stacking between the phenyl rings. The polarized protons of the p-nitrophenyl ring serve as donors to the electron-rich π-systems of adjacent molecules, forming a “herringbone-like” aromatic zipper that stabilizes the hydrophobic layers in the ac plane.

The introduction of the tyrosine residue in the amino acid molecule was found to alter the supramolecular assembly, as the dipeptide Boc-pNPhe-Tyr crystallizes as a monohydrate in the same chiral space group P2_1_. The water molecule present in the structure acts as a bridge, participating in a hydrogen-bonding network between the tyrosine hydroxyl groups of neighboring columns through intermolecular interactions.

While the monomeric Boc-pNPhe self-assembles into 2D hydrogen-bonded layers, the *p*-nitro free dipeptide Boc-L-phenylalanyl-tyrosine (Boc-Phe-Tyr) organizes into a 3D helical columnar architecture. This structural shift is driven by the additional hydrogen-bonding capacity of the peptide bond and the tyrosine hydroxyl group, which favor the formation of the tetragonal network over the planar sheets of the monomer. In addition, while related Boc-L-phenylalanyl-phenylalanine dipeptide forms hexagonal nanotubes, Boc-Phe-Tyr adopts a tetragonal symmetry crystallizing in space group P4_3_. Both systems form channel-type architectures with a characteristic ~5.2 Å stacking axis.

Moreover, the determination of the crystal structures of Boc-Phe-Tyr and Boc-pNPhe-Tyr helps to demonstrate the influence of pNPhe and Tyr residues. The first dipeptide forms a tubular channel-type architecture with 4-fold symmetry, similar to the six-fold channel-type tubular symmetry found in Phe-Phe crystals. However, the dipeptide Boc-pNPhe-Tyr displays a monoclinic symmetry, point group 2, also exhibited by the crystal structure of Boc-pNPhe-pNPhe.

Further studies on dynamic light scattering, optical absorption, and photoluminescence under different experimental conditions, such as temperature, solvents and pH, need to be addressed to deepen the understanding of the self-assembly process. Also, the growth of suitable crystals under those different experimental conditions and a full determination of their respective crystal structure will help to establish a fuller comprehension of self-assembly in these remarkable systems.

## Figures and Tables

**Table 1:** Maximum OA and PL wavelengths for Boc-pNPhe, Boc-pNPhe-Tyr, Boc-Phe-Tyr, Boc-Phe-Phe and Boc-pNPhe-pNPhe in ethanol solutions.

Dipeptide	λ_abs_ [nm]	λ_exc_ [nm]	λ_emi_ [nm]	Red Shift [nm]
Boc-pNPhe	275	270; 280	315; 326	45; 46
Boc-pNPhe-Tyr	255; 275; 282 353; 368; 387	275; 282 350; 360	325; 339 416; 432	50; 57 66; 72
Boc-Phe-Tyr [18]	252; 258; 265; 269; 278; 285	278	306	28
Boc-Phe-Phe [17]	248; 253; 259; 265	259	285	26
Boc-pNPhe-pNPhe [18]	268; 272; 275; 279	272	320; 357	48; 85

## Data Availability

The original contributions presented in this study are included in the article/Supplementary Materials. Further inquiries can be directed to the corresponding authors.

## Supplementary Materials

The following supporting information can be downloaded at: https://www.mdpi.com/article/10.3390/ma19071319/s1.
