# Supplementary material for: Self-Assembly and Crystal Structure of Boc-Protected Dipeptides Containing L-Phenylalanine and L-Tyrosine
Source: Materials (Basel). 2026 Mar 26;19(7):1319. doi: 10.3390/ma19071319 (PMC13073371; doi:10.3390/ma19071319)
Supplement: Supplementary file 1 [file materials-19-01319-s001.zip › materials-4185588-supplementary.pdf]

Self-assembly and crystal structure of Boc-protected dipeptides containing L-phenylalanine and L-tyrosine

Rosa M. F. Baptista [1,*], Alejandro P. Ayala[2], Clara S. B. Gomes [3], Daniela Santos [1], Michael S. Belsley [1] and Etelvina de Matos Gomes [1,*]

Supplementary Information (SI)

SI Section S1.1 *Structural Characterization by NMR Spectroscopy*

All the intermediates were characterized by NMR spectroscopy on a Bruker Avance III 400 at an operating frequency of 400 MHz for ^{1}H and ^{13}C, respectively. The solvent used to prepare the samples was DMSO-d_6 (dimethylsulfoxide >99.80 atom% D). Chemical shifts were reported in parts per million and tetramethylsilane (TMS) was used as an external reference. ^{1}H NMR (400 MHz, DMSO-d_6, ppm) δ 1.10 (3 × CH_3-Boc, s, 9H), 2.59 (CH_2-β, m, 1H), 2.71 (CH_2-β, m, 2H), 3.09 (CH_2-β, m, 1H), 4.02 (CH-α, m, 1H), 4.25 (CH-α, m, 1H), 5.92 (NH, d, 1H), 6.36 (2 × CH-Tyr, d, 2H), 6.64 (2 × CH-Tyr, d, 2H), 7.23 (2 × CH-pNPhe, d, 2H), 7.71 (2 × CH-pNPhe, d, 2H), 8.08 (OH, broad s, 1H), 8.35 (NH, d, 1H); ^{13}C NMR (100.6 MHz, DMSO-d_6, ppm): δ 27.39, 32.56, 37.33, 53.53, 54.39, 77.19, 115.00, 122.27, 122.59, 130.32, 141.87, 144.94, 145.67, 146.27, 155.34, 156.63, 169.86, 171.79. The NMR spectroscopic data presented in this section correspond specifically to the newly synthesized compound Boc-pNPhe-Tyr, as this dipeptide has not been previously reported, unlike Boc-Phe-Tyr, whose synthesis and characterization are available in the literature [18].

SI Section S1.2. Crystal Data

Table S1. Crystallographic data and refinement parameters for the amino acid Boc-pNPhe and the dipeptides Boc-Phe-Tyr and Boc-pNPhe-Tyr.

	**Boc-pNPhe**	**Boc-Phe-Tyr**	**Boc-pNPhe-Tyr**
CCDC number	2529055	2529054	2530636
Empirical formula	$C_{14}H_{18}N_2O_6$	$C_{23}H_{27}N_2O_6$	$C_{23}H_{29}N_3O_9$
Formula weight	310.30	427.46	490.48
Temperature [K]	100.00	300.00	293(2)
Crystal system	monoclinic	tetragonal	Monoclinic
Space group (number)	$P2_1$ (4)	$P4_3$ (78)	$P2_1$ (4)
a [Å]	10.7575(6)	21.2134(13)	11.050(2)
b [Å]	5.1460(3)	21.2134(13)	5.0136(9)
c [Å]	13.4066(8)	5.1784(5)	22.712(4)
α [°]	90	90	90
β [°]	91.739(4)	90	102.348(6)
γ [°]	90	90	90
Volume [Å^3]	741.82(7)	2330.3(4)	1229.1(4)
Z	2	4	2
ρ_{calc} [gcm^{-3}]	1.389	1.218	1.325
μ [mm^{-1}]	0.928	0.088	0.103
F(000)	328	908	518
Crystal size [mm^3]	0.213×0.034×0.026	0.12×0.05×0.04	0.02x0.08x0.50
Crystal colour	clear light colourless	clear light colourless	Clear light colourless
Crystal shape	block	prism	Needle
Radiation	CuK_α (λ=1.54178 Å)	MoK_α (λ=0.71073 Å)	MoK_α (λ=0.71073 Å)
2θ range [°]	8.22 to 144.70 (0.81 Å)	5.43 to 50.86 (0.83 Å)	5.51 to 51.36 (0.8/ Å
Index ranges	-13 ≤ h ≤ 13<br>-5 ≤ k ≤ 6<br>-16 ≤ l ≤ 16	-25 ≤ h ≤ 25<br>-25 ≤ k ≤ 25<br>-6 ≤ l ≤ 6	-13 ≤ h ≤ 13<br>-6 ≤ k ≤ 6<br>-27 ≤ l ≤ 27
Reflections collected	20560	60843	45623
Independent reflections	2864<br>R_{int} = 0.0930<br>R_{sigma} = 0.0427	4313<br>R_{int} = 0.1084<br>R_{sigma} = 0.0457	4630<br>R_{int} = 0.5276<br>R_{sigma} = 0.2883
Completeness	99.4 %	99.8 %	99.8%
Data / Restraints / Parameters	2864/1/205	4313/322/271	4630/2/323
Goodness-of-fit on F^2	1.085	1.247	0.938

Final *R* indexes [$I \geq 2\sigma(I)$]	R_1 = 0.0893<br>wR_2 = 0.2141	R_1 = 0.1193<br>wR_2 = 0.1805	R_1 = 0.0876<br>wR_2 = 0.1864
Final *R* indexes [all data]	R_1 = 0.1078<br>wR_2 = 0.2285	R_1 = 0.1859<br>wR_2 = 0.2075	R_1 = 0.1948<br>wR_2 = 0.2155
Largest peak/hole [eÅ^{-3}]	0.34/-0.33	0.24/-0.24	0.26/-0.24

Table S2. Intermolecular hydrogen bond geometry for Boc-pNPhe (Å, °)

D-H...A	d(D-H)	d(H...A)	d(D...A)	<(DHA)	Symmetry Code
O4-H4...O1	0.84	1.97	2.807(9)	176	(i)
N1-H1...O5	0.88	2.36	3.063(9)	138	(ii)
C5-H5...O3	0.95	2.54	3.460(10)	164	(iii)

Symmetry codes: (i) x-1, y-1, z; (ii) x, y-1, z; (iii) -x, y+1/2, -z+2.

Table S3. Intermolecular hydrogen bond geometry for Boc-Phe-Tyr (Å, °)

D-H...A	d(D-H)	d(H...A)	d(D...A)	<(DHA)	Symmetry Code
O1-H1A...O4	0.82	1.91	2.729(10)	174	(iv)
N2-H2...O5	0.86	2.18	2.950(10)	149	(v)
C11-H11...O4	0.93	2.59	3.252(12)	129	(iv)

Symmetry codes: (iv) 1-y, x, z+3/4; (v) x, y, z-1.

Table S4. Intermolecular hydrogen bond geometry for Boc-pNPhe-Tyr (Å, °)

D-H...A	d(D-H)	d(H...A)	d(D...A)	<(DHA)	Symmetry Code
C1-H1C...O2	0.96	2.41	3.007(11)	119.7	
C4-H4B...O2	0.96	2.30	2.912(10)	121.2	
O6-H6...O7	0.82	1.97	2.671(7)	143.7	(vi)
N1-H100...O2	0.90(3)	2.15(4)	3.013(9)	161(9)	(vii)
N2-H200...O5	0.91(3)	2.14(5)	2.968(9)	151(8)	(viii)
O9-H1O9...O8	0.8443	1.947	2.722(9)	152.27	
O9-H2O9...O8	0.8587	2.223	3.081(9)	177.0(2)	(vii)

Symmetry codes: (vi) -x+1,y-1/2,-z+1; (vii) x,y+1,z; (viii) x,y-1,z
